# Cardiac Protection by Preconditioning Is Generated via an Iron-Signal Created by Proteasomal Degradation of Iron Proteins

**DOI:** 10.1371/journal.pone.0048947

**Published:** 2012-11-14

**Authors:** Baruch E. Bulvik, Eduard Berenshtein, Esther G. Meyron-Holtz, Abraham M. Konijn, Mordechai Chevion

**Affiliations:** 1 Departments of Cellular Biochemistry and Human Genetics, Faculties of Dental Medicine and Medicine, The Hebrew University of Jerusalem, Jerusalem, Israel; 2 Human Nutrition and Metabolism, Faculties of Dental Medicine and Medicine, The Hebrew University of Jerusalem, Jerusalem, Israel; 3 Laboratory for Molecular Nutrition, Faculty of Biotechnology and Food Engineering, Technion - Israel Institute of Technology, Technion City, Haifa, Israel; Virginia Commonwealth University Medical Center, United States of America

## Abstract

Ischemia associated injury of the myocardium is caused by oxidative damage during reperfusion. Myocardial protection by ischemic preconditioning (IPC) was shown to be mediated by a transient ‘iron-signal’ that leads to the accumulation of apoferritin and sequestration of reactive iron released during the ischemia. Here we identified the source of this ‘iron signal’ and evaluated its role in the mechanisms of cardiac protection by hypoxic preconditioning. Rat hearts were retrogradely perfused and the effect of proteasomal and lysosomal protease inhibitors on ferritin levels were measured. The iron-signal was abolished, ferritin levels were not increased and cardiac protection was diminished by inhibition of the proteasome prior to IPC. Similarly, double amounts of ferritin and better recovery after *ex vivo* ischemia-and-reperfusion (I/R) were found in hearts from *in vivo* hypoxia pre-conditioned animals. IPC followed by normoxic perfusion for 30 min (‘delay’) prior to I/R caused a reduced ferritin accumulation at the end of the ischemia phase and reduced protection. Full restoration of the IPC-mediated cardiac protection was achieved by employing lysosomal inhibitors during the ‘delay’. In conclusion, proteasomal protein degradation of iron-proteins causes the generation of the ‘iron-signal’ by IPC, ensuing *de-novo* apoferritin synthesis and thus, sequestering reactive iron. Lysosomal proteases are involved in subsequent ferritin breakdown as revealed by the use of specific pathway inhibitors during the ‘delay’. We suggest that proteasomal iron-protein degradation is a stress response causing an expeditious cytosolic iron release thus, altering iron homeostasis to protect the myocardium during I/R, while lysosomal ferritin degradation is part of housekeeping iron homeostasis.

## Introduction

Ischemia/reperfusion (I/R) injury is a common event underlying many pathological conditions, including coronary heart diseases, lung transplantation and brain disorders [1], [2]. Reperfusion, subsequent to prolonged ischemia is essential for survival but may cause additional tissue damage [3]. Thus, protecting against ischemia and reoxygenation-associated injuries during surgery or acute infarction is a continuous challenge.

Ischemic preconditioning (IPC) is a protective procedure accomplished by exposing the organ to a minor stress, which by itself does not cause noticeable harm. In cardiac IPC, the heart is subjected, to short ischemic episodes separated by short perfusion periods rendering the myocardium more tolerant to subsequent prolonged (and damaging) ischemia [4]. IPC reduces infract size, maintains elevated levels of high energy phosphate bonds and accelerates the recovery of hemodynamic activity of the heart [5]. It is widely accepted that IPC mitigates the reperfusion injury. Though, extensively studied, heart protection by IPC is not fully understood.

Due to deprivation of oxygen during ischemia, oxidative phosphorylation is terminated and glycolysis is activated triggering the accumulation of lactic acid and intracellular acidification, a drop of ATP levels and increased demand of the beating heart for energy which cannot be met, leading to cessation of heart function [6]. Reactive oxygen-derived species (ROS), including free radicals, are produced at the onset of reperfusion and contribute to tissue damage, and are considered as major contributors to I/R injury [7]. ROS formation is amplified by newly mobilized labile and redox-active iron ions, through the Fenton/Haber-Weiss reactions [8], [9], [10], [11], [12], [13]. These events, together with uncontrolled elevation of intracellular {Ca^2+^] in early reperfusion lead to a marked decline in tissue integrity, which is associated with activation of degradation enzymes and compromised ATP-dependent repair processes [6].

Ferritin is the major cellular iron storage and detoxifying protein. Its synthesis is under tight translational regulation. mRNA levels of ferritin subunits are continuously present in cells. Their regulation involves two repressor iron regulatory proteins (IRPs) which register cytosolic iron concentrations, and when depleted they attach to an iron responsive element (IRE) on the 5′end of ferritin-subunits mRNA [14].

Previous findings from our lab, suggest that a short burst of labile iron could serve as a cellular trigger for protective mechanisms in heart IPC, which in turn, would reduce the damage caused at early reperfusion following prolonged ischemia [15], [16]. During ischemia iron is mobilized and re-distributed [13]. Throughout the IPC procedure, minute amount of iron is mobilized and serves as an ‘iron signal’, for rapid ferritin synthesis. Within 15 min of the IPC procedure ferritin level reached several-fold its basal value at stabilization. During subsequent prolonged ischemia a relatively large amount of labile iron is mobilized and sequestered by the newly synthesized apo-ferritin, and kept in a redox-inactive state, thus, the tissue is protected from reperfusion injury. Simulation of endogenous iron mobilization by providing exogenous iron (without applying the IPC procedure) resulted in increased ferritin levels and cardio-protection. Conversely, when the ‘iron signal’ was inhibited by iron-selective chelators, the IPC-mediated protection was eliminated [17].

Apparently, the ‘iron signal’ could stem from, at least, three sources: (i) Heme catabolism by Heme-oxygenases (HO), (ii) degradation of Fe-S clusters and (iii) degradation of iron-containing proteins, mainly ferritin.

The amount of iron released from iron-sulfur clusters is probably too low for the formation of the iron signal. However, myoglobin and the respiratory cytochromes are abundant in cardiac tissue, and upon their breakdown yield heme, which can be catabolized by HO thus, ensuing the release of labile iron. HO-1 and HO-2 are ubiquitously expressed and active in the heart and could serve as the source for labile iron throughout the IPC. Indeed, HO activity is affected by various types of stress [18], [19], [20]. HO-1 expression is induced by heme and by various non-heme substances. The duration of the IPC procedure might be too short for complete expression of HO-1. HO-2 activity could be modified by the cellular redox status, which in turn, could play a key role in the generation of the ‘iron signal’ [21]. Thus, HO-2 could lead to rapid iron accumulation without the need for its *de novo* synthesis.

Ischemia generates conditions promoting elevated levels of intracellular-damaged proteins, which are degraded by the ubiquitin-proteasome system (UPS) [22]. Some UPS subunits, like many other cellular proteins, lose their activity during I/R and turn into oxidized and ubiquitinated proteins [23]. Using the rat model of cardiac I/R injury, it was shown that a selective, rather than a global, proteasome inhibition occurred, which suggested that some specific proteasome functions remained active [24]. Indeed, ferritin can be degraded by both lysosomal proteolysis [25], [26] and the proteasome pathway [27].

The aims of this paper were to determine the source of the iron for the ‘iron signal’ in IPC and to evaluate its role in mechanisms of cardiac protection by hypoxic preconditioning as well. We verified the predominant origin of this iron as ferritin; the mechanism was by ferritin degradation by the proteosome causing the release of its iron thus, inducing *de novo* apoferritin synthesis, leading, in turn, to the chelation of the labile, redox active, iron which was released during the ischemia and thus, protecting the heart. We discovered that the same mechanisms are true for both *ex vivo* IPC and in* vivo* hypoxic preconditioning.

## Materials and Methods

Detailed methods are available as Supporting Information.

### Animals and Experimental Design

Male rats of the Sprague–Dawley strain (weighing 250–300 g) were used throughout. All the experimental protocols were approved by the ‘Institutional Animal Care and Use Committee’ of the Hebrew University of Jerusalem, conforming to the Guide for the Care and Use of Laboratory Animals published by the U.S. National Institutes of Health (NIH Publication No. 85–23, revised 1996).

### Perfusion Protocols

Rats were anesthetized and their hearts mounted on the Langendorff apparatus [13]. The basic experimental protocols included 10 min stabilization, 35 min global ischemia and 60 min. reperfusion (I/R). The IPC procedure included 3 cycles of 2 min global ischemia separated by 3 min perfusion, altogether 15 min. The stabilization period was extended to 25 min in hearts subjected to I/R (without IPC)in order to compensate for the duration of the IPC procedure. Hemodynamic parameters were monitored throughout the entire experiment. At pre-determined time points along the protocols, hearts were frozen rapidly in liquid nitrogen and kept at −80°C until analyses of the biochemical parameters. These were measured, at the completion of the IPC procedure, at the end of the ischemia and at the end of the reperfusion period, in specimens taken from the left ventricle [28]. The hemodynamic parameters included the left ventricular peak systolic pressure (PSP), end diastolic pressure (EDP), developed pressure (DP = PSP−EDP), heart rate (HR), work index (WI = DP×HR) and ±dp/dt_max_. Cardiac recovery was calculated, relative to the stabilization phase (controls), at the end of the experiment protocol.

### Experimental Protocols

**Figure 1:**
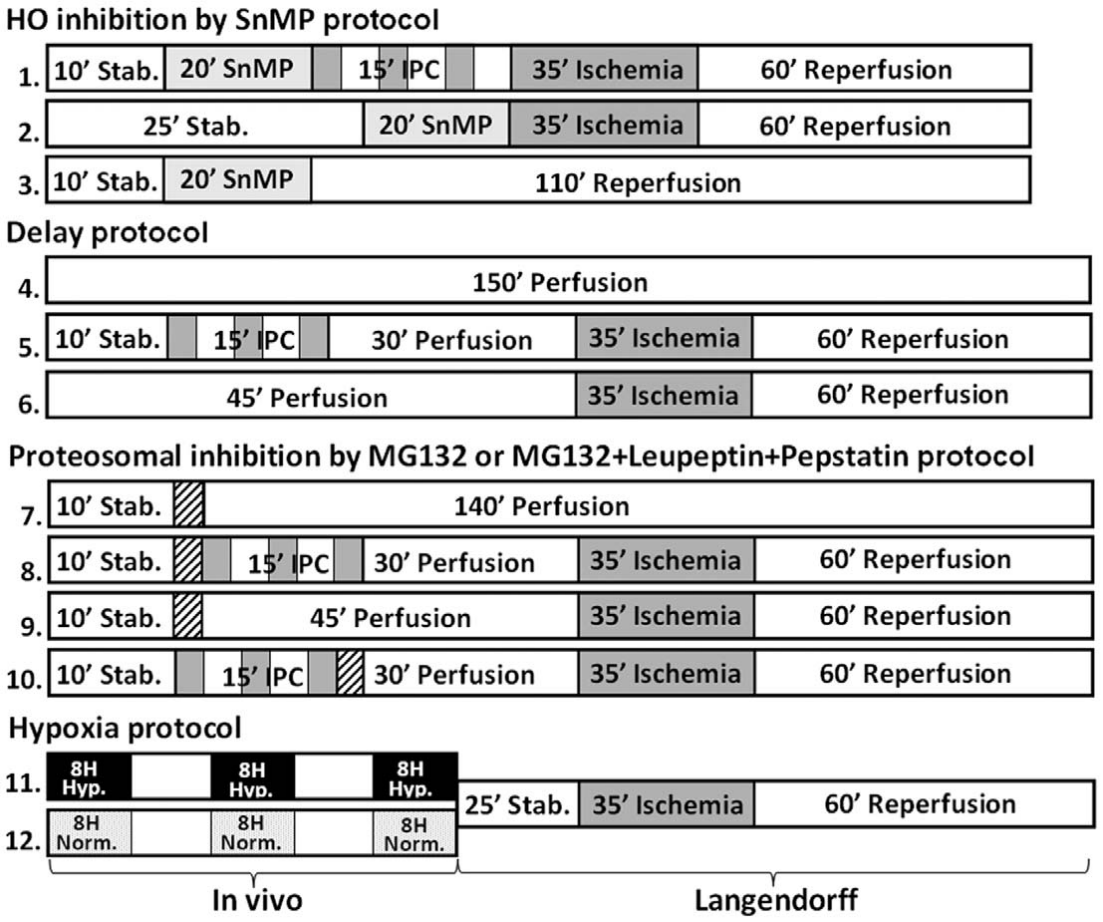
Schematic illustration of the protocols for the experimental groups. Ischemia- dark gray background; 3 min during IPC or 35 min in the prolonged ischemia. Protease inhibitor cocktails- black oblique lines; 3 min before or after IPC. Heme-oxygenase inhibitor, SnMP - light gray background, 20 min before IPC. In vivo hypoxia (9% O2)- black background 8 h per day for 3 days.

Experimental protocols were performed in groups as depicted in figure 1.

#### Inhibition of Heme-oxygenase activity

HO was inhibited with tin-mesoporphyrin-dichloride (SnMP, Frontier Scientific, Logan, UT, USA). Following stabilization, hearts were perfused with 20 µM SnMP for 7 min (100 ml perfusate) with a constant flow of 14 ml/min SnMP followed by 13 min of a “washing out” period. The hearts were then perfused for 110 min with Krebs-Henseleit Buffer (KH-buffer). Alternatively, the I/R procedure was executed with or without prior IPC (groups 3, 1 and 2 respectively).

#### ‘Delay’ of I/R

Following IPC hearts were perfused for an additional 30 min, delaying the I/R part of the protocol (‘delay’ in group 5). The control hearts (I/R-without prior IPC or uninterrupted perfusion, groups 4 and 6 respectively) were also delayed by 30 min; thus, these hearts were perfused for a total of 150 min.

#### Inhibition of the proteasomal and/or the lysosomal protein degradation pathways before or after the IPC procedure

These were achieved by the use of the proteasome inhibitor MG132 (Sigma-Israel) (6 µmol/L) or a combination of lysosomal protease inhibitors containing leupeptin and pepstatin-A (Sigma-Israel) (23.4 µmol/L and 8.8 µmol/L respectively _ = _ dose1) for 3 min, before or after the IPC. A cocktail of all three inhibitors was used for inhibiting total cellular proteolytic activity (groups 8 and 10).

Control hearts were perfused with the inhibitors for 3 min followed by 140 min, or for 45 min perfusion followed by the I/R protocol (but without IPC, groups 7 and 9).

#### Hypoxia-induced preconditioning of the rat

Rats were daily exposed for 8 h to hypoxia (9% O_2_, isobaric pressure) for 3 consecutive days [29]. Their hearts subjected to the *ex vivo* I/R protocol in the Langendorff apparatus. The hearts were perfused for 25 min followed by I/R. The controls were hearts from normoxic rats (groups 11 and 12).

### Biochemical Assays

*Cytosolic heart ferritin* was measured by ELISA as previously described [16].

#### Mean Iron content in ferritin and calculation of the average number of iron atoms per ferritin molecule

Ferritin was immune-precipitated. The precipitate was dissolved in nitric acid and its iron content measured spectrophotometrically with bathophenantroline-disulphonate [30].

*Electro Mobility Shift Assay (EMSA) of IRPs* was carried out under low oxygen pressure [31], [32].

*Ferritin mRNA quantification by real-time qPCR (qRT-PCR)* was done according to previously published protocols [33]. The change in gene expression relative to 25 min un-interrupted perfusion was normalized to β-actin and calculated using the 2^−ΔΔCT^ method [34].

### Statistical Analysis

Comparison between values for the same group at various time points along the experiment were analyzed by ANOVA with repeated measurements. Differences in variables between groups for a specific time point were analyzed using one-way ANOVA, followed by the Scheffe post hoc test for multiple comparisons (with p<0.05).

## Results

Improved heart hemodynamics following IPC+I/R is a known phenomenon which is well documented and explained by virtue of several mechanisms [5], [6].

Recently we proposed that cardio-protection by IPC involves the generation of an ‘iron signal’ and accumulation of cellular ferritin [17]. The origin for the ‘iron signal’ remained unidentified. Among several possibilities, enhanced degradation of heme by increased activity of heme-oxygenase (HO) was considered.

We used Sn-Mesoporphyrin (SnMP), a potent competitive inhibitor of HO [35], to examine this possibility. After stabilization, a non-toxic dose of 20 µmol/L SnMP was added to the perfusion medium (KH-buffer), before the I/R procedure, with or without prior IPC (Figure1, groups 1–3, and Table 1). Perfusing a total 2 µmole of SnMP led to a noticeable enhanced recovery of the work index (WI), both for I/R and IPC+I/R protocols (Table 1). The values were similar to those accomplished by IPC+I/R (without SnMP), which were 70% of the initial WI value [17]. SnMP did not abolish ferritin accumulation following IPC, which attained a level of (0.38±0.06) µg/mg protein, an 88% increase over the basic level of 0.22±0.06 µg/mg protein (N = 4). Though, SnMP inhibited HO activity, it did not eliminate the ‘iron signal’. The presence of SnMP decreased iron release from heme proteins, nevertheless the heart was protected against I/R even without IPC. Heart rate (HR) recovered considerably in the presence of SnMP, however hearts did not completely relax, as indicated by the EDP and the declined DP values (Table 1). Thus, we concluded that even though heme protected the heart it is not the primary source for the ‘iron signal’.

**Table 1:** Hemodynamic recovery of hearts receiving inhibitors of specific enzymes before IPC.

		dose	HR (%)	EDP (mmHg)	Dp (%)	+dp/dt (%)	WI (%)
SnMP	Perf. 25′ (3)	2 µmole	100±2	−10±3	86±8	103±10	86±8
	I/R (6)	2 µmole	84±5*	36±6*	71±7*	103±13	60±7*
	IPC+I/R (6)	2 µmole	87±6*	27±12*	76±9	95±18	66±9*
Proteases Cocktail	Perf. 25′	1	78±0	0±7	94±7	76±7	73±6
	I/R	1	87±11	2±4	70±10	77±8	59±8
	IPC+I/R	1	111±9*	1±7	73±6	90±10	81±9
		0.5	109±4	10±6	74±6	87±6	81±5
		0.25	89±5	10±9	83±10	99±9	73±5
MG132	Perf. 25′	3 µmol/L	110±10	9±5	79±9	77±9	85±3
	I/R	3 µmol/L	70±8*	50±14*	51±9	75±15	35±6*
	IPC+I/R	3 µmol/L	81±8*	45±4*	50±3*	77±27	40±5*

Hemodynamic parameters were calculated at completion of the reperfusion: for perfusion only, I/R and IPC+I/R. (For experimental protocols see Figure 1 groups 1–3 and 7–9).

SnMP- hemodynamic recovery of hearts receiving 2 µmole of the HO inhibitor SnMP.

Proteases cocktail- hemodynamic recovery of hearts receiving a cocktail of proteasome *and* lysosomal proteases inhibitors. Dose 1 of the cocktail was composed of MG132, 6 µmol/L; leupeptin 23.4 µmol/L; pepstatin-A 8.8 µmol/L.

Proteasome inhibitor- hemodynamic recovery of hearts receiving 3 µmol/L of the proteasome inhibitor MG132.

The numbers in parentheses indicate the number of repetitious experiments. Controls (considered 100%) are the heart’s hemodynamic parameters at the end of a 10 min perfusion period (end of the stabilization period).

Results are means±SE.

* Significantly different from controls-perfusion only (p<0.05).

Protein degradation pathways were studied to further investigate the source for the ‘iron signal’. We used MG132 for inhibiting the proteasomal pathway and Leupeptin and Pepstatin-A for inhibiting lysosomal protein digestion (Figure1, groups 7–9) [36]. The full dose of inhibitors applied to hearts subjected to continuous perfusion showed a small anti-inotropic effect and a relative decline of the WI to 73% (Table 1). A cocktail of the inhibitors administered to hearts preceding the I/R or IPC+I/R showed a noticeable protection of the WI when compared to hearts that did not receive the inhibitors (Tables 1 and 2). However, ferritin did not accumulate and remained approximately 0.22 µg/mg protein. A lower dose of the cocktail of inhibitors had no clear effect but there was a trend toward a limited cardiac protection. Consequently, we studied the function of the proteasome and the lysosomal pathways, separately. The use of MG132, at a concentration of 3 µmol/L, caused exacerbation of the recovery of the WI, after IPC+I/R (Tables 1 versus 2). Ferritin heart levels for either the perfusion control and at the end of IPC were comparable (0.22±0.01 and 0.23±0.03 µg/mg protein, respectively). Repressed DP recovery was the main reason for the limited recovery of the WI, due to high EDP values, indicative for diastolic dysfunction.

MG132 administered prior to IPC abolished the IPC-induced protection (Table 1). MG132 inhibited the degradation of cytosolic iron-containing proteins, mainly ferritin, and the release of their iron to the labile iron pool (LIP) and thus, prevented the induction of *de novo* apoferritin synthesis.

**Figure 2:**
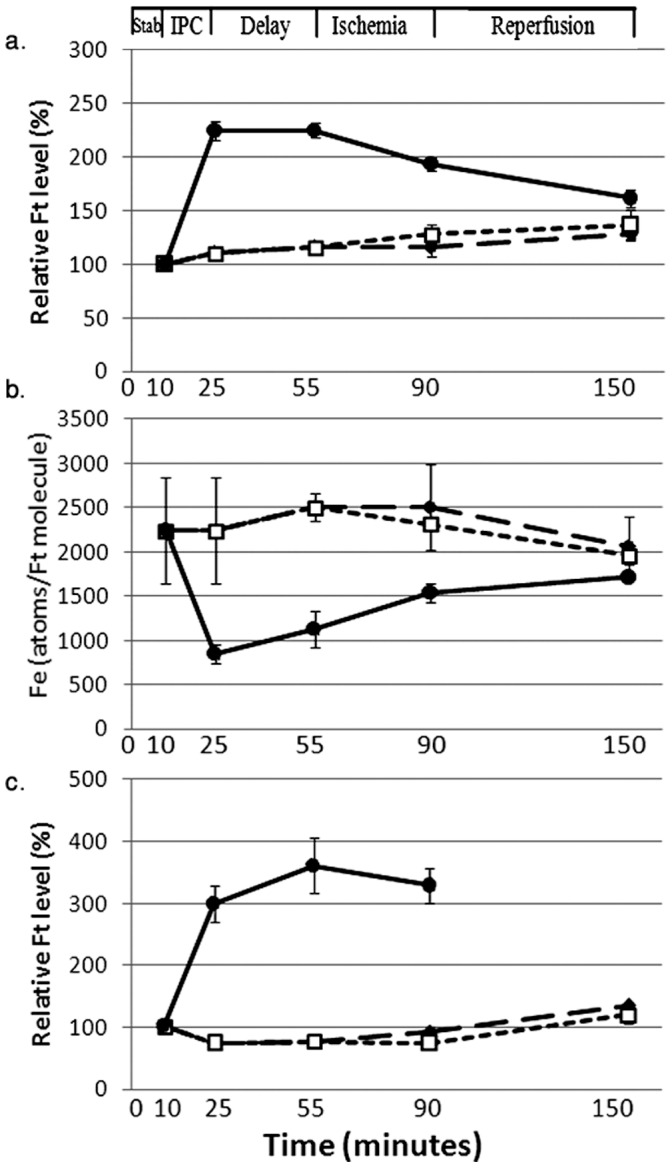
Ferritin and ferritin-bound iron levels in hearts subjected to I/R. Ferritin and ferritin-bound iron levels with or without prior IPC and a ‘delay’ period. a. Relative ferritin protein levels (% of the post stabilization period). b. Ferritin-bound iron (number of iron atoms per molecule of ferritin). c. Relative ferritin protein levels (% of the post stabilization period) in hearts infused with a cocktail of protease inhibitors (MG132-3 µmol/L, leupeptin-11.7 µmol/L, pepstatin-A-4.4 µmol/L): perfusion -black diamond, IPC+‘delay’ +I/R - black circle, ‘delay’+I/R- white square. Results are means±SE of 6 experiments. SE for perfusion and ‘delay’+I/R groups are too small to be seen.

Ferritin plays a central role in heart protection by IPC [17]. Immediately following the IPC procedure we inserted a ‘delay’ stage, which consisted of a 30 min period of un-interrupted perfusion. It was contemplated that the ‘delay’ would lead to improved heart pre-condition thus, subsequently better I/R performance, concomitantly with increased levels of cardiac ferritin (Figure1, groups 4–6). However, this delay did not improve functional protection but rather proved moderately detrimental compared to the results of previous experiments, which did not include a ‘delay’ stage (Table 2) [17]. IPC stimulated ferritin accumulation and it remained at a constant high level during the following ‘delay’ period. During the subsequent, prolonged, ischemia period, ferritin declined moderately, and remained above the basal concentration (Figure 2A). The number of iron atoms within a ferritin molecule was inversely related to the levels of ferritin (protein). Ferritin molecules were less saturated, enabling ferritin to sequester additional labile iron ions (Figure 2B). Ferritin subunits mRNAs, for both H and L, did not change during the entire’ delay’ protocol (data not shown).

**Table 2:** Recovery of hemodynamic parameters of hearts subjected to perfusion, IPC+‘delay’+I/R, and I/R with or without protease inhibitors.

		HR (%)	EDP (mmHg)	Dp (%)	+dp/dt (%)	WI (%)
Delay	Perfusion	98±2	0.0±0.0	96±9	93±11	96±9
	I/R	86±5*†	49±9*†	40±16*†	52±14*†	35±14*†
	IPC+I/R	96±3	30±13*	60±10*	74±4*	58±10*
Proteasesinhibitors	inhibitor cocktail (3)	96±6	9±16	83±11	89±9	80±7
	Proteosomal inhibitor (3)	85±18	28±10	54±5*	73±7	46±6*
	Lysosomal inhibitors(4)	90±9	8±10	81±4	94±6	73±8

Delay- hemodynamic recovery of hearts undergoing I/R with or without IPC+‘delay’.

Proteases inhibitors- hemodynamic recovery for IPC+‘delay’+I/R in groups of hearts receiving: an inhibitor cocktail (leupeptin 11.7 µmol/L; pepstatin A 4.4 µmol/L; MG132-3 µmol/L), a proteasomal inhibitor (MG132-3 µmol/L), a lysosomal protease inhibitors cocktail (leupeptin 11.7 µmol/L, pepstatin-A 4.4 µmol/L). The inhibitors were applied subsequent to the IPC procedure followed by the ‘delay’ period and then subjected to I/R.

For experimental protocols see figure 1 groups 4–6 and 10. Controls (considered 100%) are the heart’s hemodynamic parameters at the end of a 10 min perfusion period (end of the stabilization period). Results represent means±SE. The numbers in parentheses indicate the number of repetitious experiments.

* Significantly different from controls-perfusion only (p<0.05). † = significantly different from IPC+I/R (p<0.05).

The marked degradation of ferritin during the prolonged ischemic period was prevented by adding a cocktail of proteasome and lysosomal protease inhibitors to the perfusate immediately after IPC (Figure 1, group 10). Thus, an elevated ferritin level was achieved and protected the heart from I/R injury, similar to that observed by IPC. In the controls (when IPC was omitted) the inhibitor cocktail did not change the cellular ferritin level (Figure 2C).We examined whether ferritin degradation in the cytosol or in the lysosome is responsible for delay-mediated cardiac dysfunction. Therefore, following IPC, the effects of the proteasome inhibitor or the lysosomal protease inhibitors were studied. Indeed, MG132 alone, when given immediately after IPC, did not prevent the impaired recovery of DP and WI values, under the {delay+I/R] protocol. On the other hand, addition of leupeptin and pepstatin-A to the perfusate resulted in protection of myocardial function, as indicated by the recovery of DP and WI (Table 2). This is in accord with the release of iron through lysosomal degradation of ferritin consequently excessive LIP following prolonged ischemia is responsible for cardiac dysfunction. In accord with the notion that increased cellular levels of ferritin serve to protect the heart against prolonged ischemia {17], the observed inhibition of lysosomal degradation of ferritin prevented the IPC-induced release of labile iron and generation of free radical mediated cardiac damage. In consequence, the lysosomal inhibitors, but not the proteasome inhibitor, markedly reduced the rate of ferritin degradation during the delay period and subsequent ischemia, and rendered the heart more resistant to prolonged ischemia.

**Figure 3:**
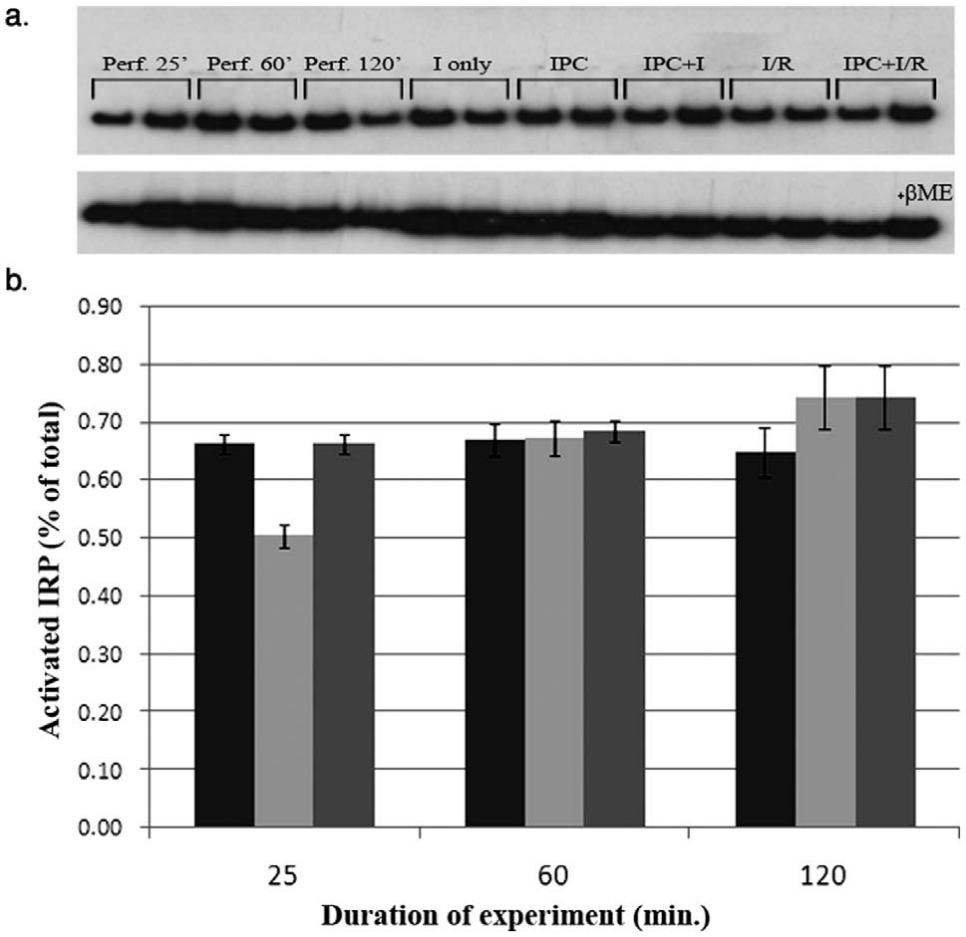
Representative EMSA using extracts of hearts subjected to I/R with and without prior IPC. a. Original phosphorimaging blots. Upper panel shows active IRPs. Lower panel displays total IRPs (+βME). b. Graphic presentation of active IRP; three scanned blots each (±SE). Perfusion only- black, I/R- gray, IPC+ I/R- light gray. No efforts were made to distinguish between IRP1 and IRP2.

The synthesis of ferritin, like some other iron proteins, is under translational control, which depends on the interaction between the iron regulatory proteins 1 and 2 (IRP 1/2) and the iron regulatory element (IRE) on the 5′ end of ferritin mRNA subunits. Thus, measuring the fraction of free *versus* IRE-bound IRPs is important [31]. This translational mode of control was already suggested to govern ferritin synthesis during the IPC phase [17]. Here, we investigated the translational regulation of ferritin by measuring the binding (activation) of the IRPs (both IRP 1 & 2) to synthetic IRE, along the I/R procedure, with and without prior IPC, by EMSA (Figure 3).Activated IRP is given as % IRP bound relatively to the total IRP, monitored in the presence of β-mercapto-ethanol.

Following IPC, the fraction of IRP that is bound to the IRE decreased by 25%, (from 66±2 to 50±2%). After the ischemia this fraction increased to the ‘normal perfusion values’ and was raised somewhat further after reperfusion. Thus, during IPC, enhanced translation of ferritin was possible. Although differential measurements for each of the IRPs (e.g. IRP1 and 2 separately) were considered we assumed that the data to be provided will not add crucial further information.

The question whether a limited cellular availability of (free) amino acids (AA) reduced the synthesis of ferritin was studied by adding a mixture of a complete set of AA (containing 1 µmol/L each). With or without the addition of AA, ferritin levels following IPC were similar, even if a delay was included (in µg ferritin/mg protein, N = 3, ±SE):

- for IPC without AA –0.43±0.06;
- for IPC with AA - 0.36±0.06; and
- for IPC+Delay and with AA - 0.39±0.09.

Thus the bio-availability of AA under IPC did not have a marked effect on ferritin levels and is not a limiting factor in its synthesis.

The ‘iron-based mechanism’ of IPC protection and the proposed role played by ferritin were evaluated by an *in vivo* rat model as well. We emulated hypoxic preconditioning in the intact rat and monitored its effect on the capacity of the heart to withstand I/R (Figure 1, groups 11 and 12).

**Figure 4:**
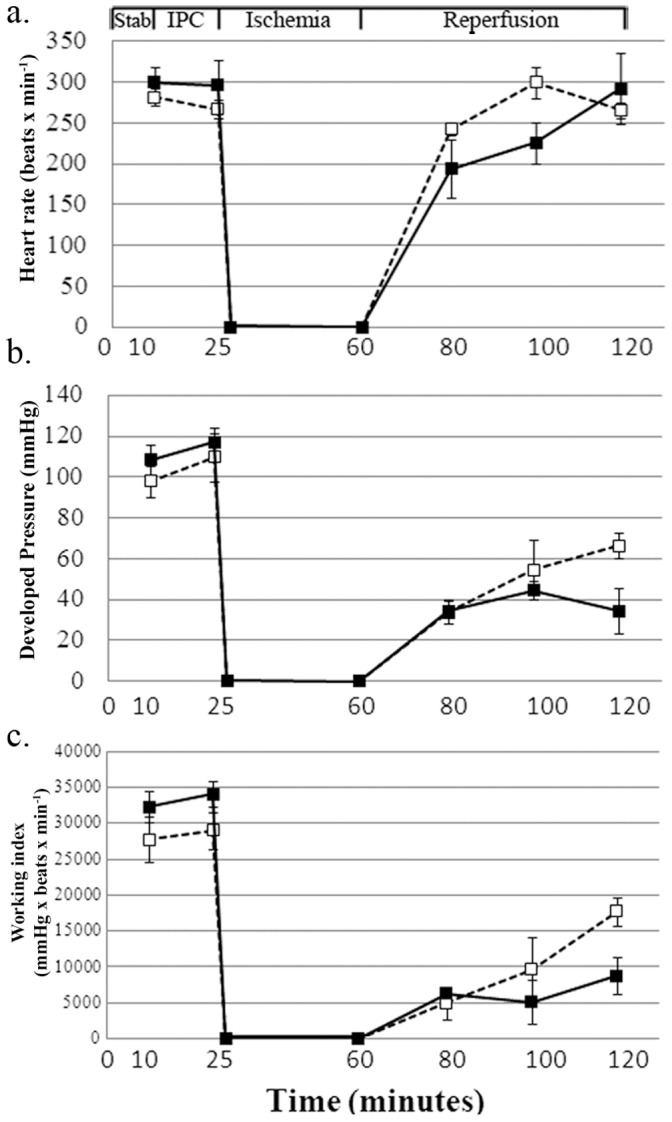
Hemodynamic parameters measured throughout the *ex vivo* procedure following *in vivo* hypoxic-preconditioning. a. Heart rate (beats per minutes). b. Developed pressure (mmHg). c. Working index (A.U. = heart rate x developed presure). Results are means±SE of 5–6 experiments. Hypoxia- white square, Normoxia- black square. Stab = stabilization period.

Cardiac hemodynamic parameters from rats undergoing I/R *ex vivo* that had already been subjected to total body hypoxia (mimicking preconditioning) recovered faster and better from prolonged ischemia then those hearts from normoxic animals (Figure 4). Similar to the *ex vivo* IPC procedure, the *in vivo* hypoxic preconditioning protected the heart against prolonged ischemia, evidently through a comparable mechanism (Table 3). Indeed, the exposure to hypoxia resulted in higher cardiac ferritin levels, as compared to hearts from control, normoxic rats (Table 3).

**Table 3:** Parameters for recovery of hearts subjected to 3 days of *in vivo* hypoxia followed by *ex vivo* I/R.

			Normoxia (4)	Hypoxia (5)	Ratio
**Hemodynamic parameters**		HR (%)	98±5	100±3	
		EDP (%)	64±7	35±8*	
		DP (%)	29±10	62±7*	
		+dp/dt (%)	90±12	76±8	
		WI (%)	27±9	62±6*	
**Ft levels**		Perfusion 25′	0.26±0.02	0.46±0.05*	1.8
		Ischemia	0.29±0.03	0.49±0.05*	1.7
		Reperfusion	0.23±0.05	0.35±0.02*	1.5
**mRNA levels**	**Ft-H**	Perfusion 25′	0.94±0.07	1.15±0.11	
		Ischemia	1.24±0.08	1.23±0.16	
		Reperfusion	1.33±0.33	1.50±0.18	
	**Ft-L**	Perfusion 25′	0.82±0.11	1.50±0.24*	
		Ischemia	1.06±0.10	1.39±0.16	
		Reperfusion	0.70±0.09	1.33±0.12*	

Hemodynamic parameters; the heart’s hemodynamic parameters at the end of a 10 min stabilization period are considered 100%.

Ferritin levels given as µg/mg protein.

mRNA levels given as arbitrary units per unit β-actin.

Results are mean±SE. Numbers in parentheses represents the number of repetitious experiments.

* = Significantly different from Normoxia (p<0.05).

Under normal physiological conditions, the heavy subunit of heart ferritin is the dominant subunit. In agreement with a previous study employing an *ex vivo* IPC protocol, subsequent to hypoxia the mRNA of cardiac ferritin-L subunit was nearly doubled, while the mRNA of the H-subunit remained unchanged. Thus, the ratio of L/H increased leading to an increase in the ferritin storage capacity (Table 3) [17].

## Discussion

The generation of ROS, including free radicals, has been frequently considered a major cause of organ damage by disease, including I/R [7], [37]. Previously, we proposed an additional viewpoint for mechanisms of heart protection against prolonged ischemia, by IPC. It involves the generation of an ‘iron signal’ during the IPC procedure and a consequent accumulation of ferritin, which acts in concert to curb iron-catalyzed and free radical-mediated reperfusion injury [12], [16], [17]. Direct measurement of the ‘iron signal’ is not feasible, but we measured the indirect response to this ‘iron signal’ - namely ferritin (protein) levels.

Iron ions stored within the ferritin molecule are redox-inactive and cannot catalyze the generation of free radicals [38], [39]. The IPC procedure causes the release of minute amounts of labile iron from proteins, thus, inducing an ‘iron signal’ [17]. Furthermore, the ‘iron signal’ leads to detachment of IRP from the IRE, ensuing ferritin translation indeed Following IPC, the fraction of IRP that is bound to the IRE decreased but after the ischemia it returned to the pre-IPC values. Thus, during IPC, enhanced translation of ferritin was possible.

Identifying the source of the ‘iron signal’ was the prime interest of this study. We considered degradation of Fe-S clusters, enzymatic breakdown of heme by HOs and/or proteolysis of intracellular iron-containing proteins, including ferritin, as possible sources for the ‘iron signal’. Fe-S clusters including those attached to iron regulatory protein one (IRP1) are limited in the cardiac cytoplasm and can hardly be accounted for as a source for iron for the ‘iron signal’. Potentially, HO could serve as a *bona fide* source of the ‘iron signal’.Cardiac HO is abundant and OH-1 is induced by a variety of means including ROS, hypoxia and stress [18], [40]. However, heart performance was not affected by the selective HO inhibitor SnMP nor did it markedly change the accumulation of ferritin during or following IPC: ferritin accumulated to ∼2.25 times its basal level, during IPC. When SnMP was administered ferritin amassed up to 1.8 times its initial amount. However, it seems that heme was degraded to some extent and some iron was released from heme by HO during IPC, consequently this pathway had some contribution to cardiac ferritin accumulation and hence to IPC protection. Indeed, our findings indicate that HO-dependent release of labile iron contributed only up to 20% of the ferritin increase and is therefore not the main source of the ‘iron signal’ in IPC. Induction of HO was previously shown to exert protection, under acute ischemic renal failure [41]. Here, the recovery of the WI of the hearts exposed to SnMP and subsequently subjected to I/R (without IPC) was better than the analogous group without the inhibitor. It can be assumed that during the prolonged ischemia, without IPC, HO releases some labile iron which is responsible for free radical mediated reperfusion injury. Consequently, hearts subjected to I/R were better protected when HO was inhibited by SnMP.

Degradation of iron-containing proteins including ferritin could serve, potentially, as the main source for the ‘iron signal’. Breakdown of a single ferritin molecule will release into the cytoplasm >1200, or more, iron ions. Degradation of intracellular proteins is mediated by either lysosomal proteases [25], [26], [42] and/or the proteasome pathway [27], [43]. MG132 revealed some minor cardiac toxicity when included in the KH-buffer of the ‘Perfusion’ group [44]. When MG132 was given to the hearts prior to the IPC procedure, the IPC-induced protection against subsequent I/R injury was lost, and there was no ferritin accumulation. The proteasome inhibitor prevented the degradation of cytosolic iron-containing proteins, including ferritin, and the release of their iron. This prevented the ‘iron signal’ and the synthesis of new apoferritin. MG132 can inhibit proteasome mediated IRP degradation and as such reduce ferritin mRNA translation. Similarly MG132 could inhibit proteasomal degradation of oxidized ferritin as reported for the microglia cell line “RAW”, following acute oxidative stress [43].

The data confirms our hypothesis that the ‘iron signal’ stems from degradation of iron containing proteins. It also substantiates our earlier proposal that heart protection by IPC occurs in concert with ferritin accumulation during the IPC procedure.

The observed close relationship between ferritin accumulation and IPC-induced cardiac protection has an additional aspect. High levels of apoferritin are needed for effective protection at the highest risk moment – the point of transition from ischemia to reperfusion, when a sudden influx of high level of oxygen enters the ischemic region. Indeed, the IPC-induced accumulation of ferritin occurs during the IPC procedure [17].

In order to examine whether ferritin levels have reached their maximum level during the 15 min of the IPC procedure or could continue to accumulate in the period following the IPC procedure a ‘delay’ period of normal perfusion was inserted between the end of the IPC procedure and the subsequent I/R. During this ‘delay’ period ferritin level remained high and stable.

During the prolonged ischemia phase hearts subjected to IPC+delay+I/R showed moderately declined levels of ferritin, unlike hearts subjected to IPC+I/R. At the onset of reperfusion, ferritin was lower (than without the delay) and the functional protection by the IPC procedure was compromised. Administering the inhibitors of lysosomal proteases together with MG132, after the IPC (but not MG132 alone) prevented the degradation of important proteins during the ‘delay’, which in turn, led to recovery of cardiac functions after I/R. Cardiac protection was achieved by inhibiting the mobilization and redistribution of harmful labile and redox-active iron, that could catalyze the formation of ROS [13]. By treating the heart with the proteasome inhibitor MG132 during the ‘delay’ period no improvement in recovery was observed, implying that during the ‘delay’ and the subsequent prolonged ischemia, ferritin is degraded in the lysosomes.

During IPC+I/R ferritin regulation is under both transcriptional and translational control since L-ferritin mRNA was up-regulated and a decline in activated IRP levels {17 and this report]. However, when I/R was separated from the IPC by a ‘delay’, ferritin mRNA levels remained stable during the whole experiment including the ischemia (not shown), thus, apparently the time gap between the IPC and the I/R proved to be crucial for the regulation and expression of ferritin.

In order to establish whether the relevance of the *ex vivo* results experiments were conducted ascertain whether *in vivo* hypoxic preconditioning will protect the heart against prolonged ischemia as well. Indeed *in vivo* and *ex vivo* studies were akin and proved that, *in vivo* hypoxic preconditioning protected the heart against prolonged ischemia, through an analogous iron-based mechanism.

We conclude that the mechanism of IPC-induced cardiac protection involves the generation of an ‘iron signal’ by virtue of proteasomal degradation of iron containing proteins, most likely ferritin and the consequent synthesis of apoferritin. In turn, the newly synthesized apoferritin molecules sequester the labile iron mobilized during the prolonged ischemia and thus, protect the heart against the reperfusion damage. During a 30 min ‘delay’ after IPC and prolonged ischemia, ferritin breaks down by lysosomal proteases resulting in a decrease in cardiac hemodynamic recovery. Inhibition of ferritin degradation during the ischemia protects the hearts hemodynamic functions.

## Supporting Information

Supporting Information S1 **Detailed methods.**
(DOC)
