# Supplementary material for: Cardiac Protection by Preconditioning Is Generated via an Iron-Signal Created by Proteasomal Degradation of Iron Proteins
Source: PLoS One. 2012 Nov 14;7(11):e48947. doi: 10.1371/journal.pone.0048947 (PMC3498359; doi:10.1371/journal.pone.0048947)
Supplement: Supporting Information S1 — Detailed methods. (DOC) [file pone.0048947.s001.doc]

**Supporting Information**

**S1. Detailed Methods.**

**Animals**

Male rats of the Sprague–Dawley strain (250–300 g) were used throughout. They were kept under standard conditions, fed a standard rat chow diet and allowed *ad libitum* access to water and food. All the experimental protocols were approved by the ‘Institutional Animal Care and Use Committee’ of the Hebrew University of Jerusalem, conforming to the Guide for the Care and Use of Laboratory Animals published by the U.S. National Institutes of Health (NIH Publication No. 85–23, revised 1996).

Materials:

All materials were of the highest purity available.

**Perfusion protocols**

Rats were anesthetized by ketamin+ xylazin (100 and 15 mg/kg, i.p., respectively). Hearts were rapidly excised with part of the ascending aorta attached, placed in ice-cold heparinized saline. The aorta was attached to a gold cannula and the heart then perfused orthogradely on the Langendorff apparatus as previously described^[1]^. All buffers used for perfusion were freshly prepared and filtered through a 0.4mμ nitrocellulose membrane. The buffers were saturated by aeration with a gas mix containing 95% O_2_-5% CO_2_, and the pH kept at 7.4. Hearts were maintained at 37.0 ± 0.1°C throughout the experiment. The perfusate used was a modified Krebs-Henseleit-buffer, as previously described, and was circulated through the hearts by hydrostatic pressure achieved by a 850mm H_2_O column^1^.

All the ischemia protocols included: stabilization for 10 minutes (baseline parameters as a reference to the effects caused by subsequent manipulations), followed by total ischemia for 35 minutes and reperfusion for 60 minutes (I/R).

The IPC procedure was composed of 3 cycles of short global ischemia (2 minutes) separated by (re)perfusion (3 minutes), altogether 15 min. In the hearts of group I/R (without IPC) the stabilization period was extended to 25 min in order to compensate for the duration of the IPC. The hemodynamic parameters were monitored throughout the entire duration of each experiment, typically for 140 or 150 min.

Biochemical parameters were measured in heart tissue samples taken at pre-determined time: (i) at completion of the IPC procedure, (ii) at the end of the ischemia, and (iii) at the end of the reperfusion period. The hearts were then quickly frozen in liquid nitrogen and kept at −80 °C until analyzed.

Heart specimens (50–150 mg) from the left ventricle were homogenized[2]. Total protein concentration was quantified using the method of Bradford^2^.

**Ferritin analysis by ELISA**

Ferritin was isolated from livers of iron loaded rats and antiserum prepared in goats as described previously[3]. Ferritin from rat hearts were prepared as described for human placenta[4] and antibodies prepared in New Zealand white rabbits as described[5].

Heart ferritin levels in the cytosolic fraction were determined by ELISA as previously described[6,7]. In short, Goat-anti-rat-liver-ferritin was diluted in Carbonate-Bicarbonate buffer 0.1mol/L pH 9.6 (Coating buffer) and used to coat 96 wells Nunc-microELISA plates (0.2ml/well). Plates were incubated for 1h at 37^0^C followed by overnight incubation at 4^0^C. After 4 washings of the coated plates with phosphate-buffered saline (PBS) 0.02mol/L, BSA 0.1 %(w/v), Tween 20-0.05% (v/v), and NaN_3_ 0.01% (w/v) (Washing buffer), the plates were blocked with buffer containing PBS 0.02mol/L, NaN_3_ 0.1 %(w/v) and Gelatin 0.5%(w/v) for 1h at 37^0^C (0.2ml/well). After 4 washings of the blocked plates with washing buffer, 0.2 ml of samples or standards diluted with PBS containing, BSA 0.5% (w/v) and Tween 20 0.05% (w/w) (Dilution buffer) were applied to the wells and incubated for 1h at 37^0^C. After further washings as above, 0.2 ml/well of Rabbit-anti-rat-heart-ferritin serum, diluted in Dilution buffer, was added and incubated for 1h at 37^0^C. Next, after washings, 0.2 ml of a solution composed of goat-anti-rabbit- γ-globulin conjugated to β-galactosidase in Phosphate Buffer 0.01mol/L pH 7.6, NaCl 10mmol/L, BSA 0.1%, PEG 6000 4%, Mg 2mmol/L and NaN_3_ 0.1% (Conjugate buffer) was added and incubated for 1h at 37^0^C. Then, following washing as above, 0.2 ml/well of chlorophenol-red-β-d-galactopyranoside (CRPG-Roche-Diagnostics) diluted in Phosphate Buffer 0.01mol/L, pH 7.2, NaCl 10mmol/L and Mg 2mmol/L (Substrate buffer) was added and incubated until color was obtained. The results were read in a microplate reader (MR 5000 Dynatech Laboratories, Chantilly, VA, USA). A primary filter with a peak transmission at 570 nm and a secondary filter with a transmission at 620 nm were used.

**Iron content in the ferritin molecule.**

Iron was determined spectrophotometrically using the chromogenic chelator bathophenantrolin-di-sulfonic acid (BPS) reagent which is specific for ferrous iron according to Nilsson et al[8]. Aliquots of the heart homogenate and anti-Rat-Heart-ferritin antibody were mixed and incubated for 72h at 4°C. The samples were then centrifuged at 20,000 × g for 20 min, the supernatant was discarded and the pellet dissolved in 0.1ml HNO_3_ (70%, density 1.413 g/mL at 20°C) and incubated overnight at 37°C. Then, the pellet-HNO3 mix was diluted (1:1) with HCl 1.2mol/L, TCA 1% and TGA 3.75% and incubated 15min. at room temperature followed by 30min. centrifugation at 3000 rpm. Next, 0.25g BPS/100ml Na-acetate 2mol/L was added in (1:1 v/v) and incubated for 2min. and the developed color intensity measured immediately at 535nm. A calibration curve was derived from samples with known concentrations of iron. From this analysis and the measured ferritin concentration, the degree of ferritin saturation by iron was calculated and presented as the number of iron atoms per ferritin molecule: Fe (atoms/ferritin molecule) =µg Fe in the immune-precipitate**/** 56 (=atomic weight of Fe) **/**mg ferritin in the immune-precipitate**/** 480,000 (~M.W. of ferritin).

**Electro Mobility Shift Assay (EMSA) of ferritin mRNAs.**

EMSA analysis was carried out under low oxygen pressure as previously described by Haile et al[9] and Meyron-Holtz et al[10]. Immediately before the assay, the tissues were ground in liquid-N_2_ cooled mortars in an anaerobic chamber followed by 10min. lysis in a degassed lysis buffer [consisting of 10mmol/L HEPES pH 7.2, 3mmol/L MgCl_2_, 40mmol/L KCl, Glycerol 5%, NP-40 0.2%, 5mmol/L DTT, 1mmol/L AEBSF, 10µg/ml Leupeptin and CompleteTM EDTA free protease inhibitor cocktail (Roche Applied Science, Indiana)]. Nuclei and debris were removed by centrifugation at 3000g. Quantities of 12.5μg clarified lysate protein were added to a buffer (final volume 12.5μl) containing Tris-HCl 25mmol/L, pH 7.5, KCl 40mmol/L, with or without 1% β-mercaptoethanol (βME). βME activates IRP1 *in vitro*. Then, 12.5 μl of a prob cocktail was added. The prob cocktail consisted of 10% glycerol, 0.2 U/μl Super RNAsine (Ambion, Texas), 6 μg/μl yeast tRNA, 5mmol/L DTT and 20nmol/L ^32^P-labelled iron regulatory element (IRE). The samples were incubated for 5 minutes at room temperature. From the 25µl sample, 20μl were loaded onto a 10% acrylamide/TBE gel and separated by electrophoresis at 180V for 3 hours. The gel was fixed, dried and exposed to phosphorimaging. Band size and darkness were measured by ImageJ software and calculated as % of bound-IRP relative to the total IRE (+βME) in the sample.

**Ferritin mRNA quantification by real-time qPCR (qRT-PCR)**.

Measurements were done according to previously published protocols[11]. RNA was isolated with a phenol-chloroform extraction solution (Molecular Research Center, Inc, Cincinnati, OH, USA) according to the manufacture’s suggestion. Total RNA (1μg) was reverse transcribed using a high capacity cDNA reverse transcription kit (Applied biosystems, Bedford, MA, USA).

All gene products were normalized to the housekeeping gene β-Actin. Primers for β-Actin and target genes were designed using Primer3 software (from: <http://frodo.wi.mit.edu/cgibin/primer3/primer3_www> .cgi) and designed so that the forward primer in each pair was complimentary to the exon–exon boundary (e.g., 3–4 in Table 1) in order to avoid genomic DNA amplification. The Nucleotide sequences used for primer design were obtained from public data bases (GenBank). qRT-PCR was carried out with 2ng cDNA templates, in 96-well plates, with power SYBR Green PCR Master Mix using the 7500 Fast Real Time PCR System ( All reagents and apparatus for the qRT-PCR were from Applied Biosystems Pty Ltd, Bedford. MA, USA). The change in gene expression relative to non-interrupted ‘25 min perfusion’ was calculated using the 2(−ΔΔCT) method[12]. The PCR primary data were obtained in triplicates.

.

Supplemental Table.


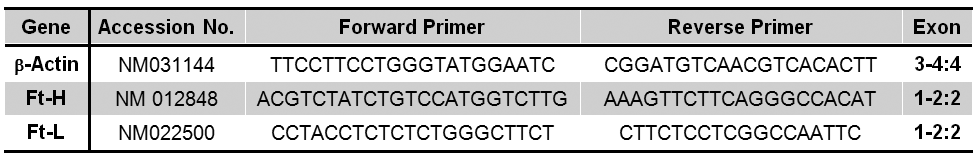
Table S1. The primers' sequences and relevant exon numbers used in the PCR assay. The nucleotide sequences used for primer design were obtained from the public database GenBank. Primers for the indicated genes were constructed using the ‘Primer3’ software, and designed so that one of the primers in each pair was complimentary to the exon-exon boundary (e.g., 3-4) in order to avoid genomic DNA amplification.

References.

1. Chevion M, Jiang Y, Har-El R, Berenshtein E, Uretzky G, et al. (1993) Copper and iron are mobilized following myocardial ischemia: possible predictive criteria for tissue injury. Proc Natl Acad Sci U S A 90: 1102-1106.

2. Drapier JC, Hibbs JB, Jr. (1996) Aconitases: a class of metalloproteins highly sensitive to nitric oxide synthesis. Methods Enzymol 269: 26-36.

3. Konijn AM, Hershko C (1977) Ferritin synthesis in inflammation. I. Pathogenesis of impaired iron release. Br J Haematol 37: 7-16.

4. Konijn AM, Tal R, Levy R, Matzner Y (1985) Isolation and fractionation of ferritin from human term placenta--a source for human isoferritins. Anal Biochem 144: 423-428.

5. Vaisman B, Santambrogio P, Arosio P, Fibach E, Konijn AM (1999) An ELISA for the H-subunit of human ferritin which employs a combination of rabbit poly- and mice monoclonal antibodies and an enzyme labeled anti-mouse-IgG. Clin Chem Lab Med 37: 121-125.

6. Berenshtein E, Vaisman B, Goldberg-Langerman C, Kitrossky N, Konijn AM, et al. (2002) Roles of ferritin and iron in ischemic preconditioning of the heart. Mol Cell Biochem 234-235: 283-292.

7. Vaisman B, Meyron-Holtz EG, Fibach E, Krichevsky AM, Konijn AM (2000) Ferritin expression in maturing normal human erythroid precursors. Br J Haematol 110: 394-401.

8. Nilsson UA, Bassen M, Savman K, Kjellmer I (2002) A simple and rapid method for the determination of "free" iron in biological fluids. Free Radic Res 36: 677-684.

9. Haile DJ, Hentze MW, Rouault TA, Harford JB, Klausner RD (1989) Regulation of interaction of the iron-responsive element binding protein with iron-responsive RNA elements. Mol Cell Biol 9: 5055-5061.

10. Meyron-Holtz EG, Ghosh MC, Rouault TA (2004) Mammalian tissue oxygen levels modulate iron-regulatory protein activities in vivo. Science 306: 2087-2090.

11. Reno C, Marchuk L, Sciore P, Frank CB, Hart DA (1997) Rapid isolation of total RNA from small samples of hypocellular, dense connective tissues. Biotechniques 22: 1082-1086.

12. Livak KJ, Schmittgen TD (2001) Analysis of relative gene expression data using real-time quantitative PCR and the 2(-Delta Delta C(T)) Method. Methods 25: 402-408.
